# PRAYAS: individual patient data meta-analysis database for Pooled Research and Analysis for Yielding Anemia-free Solutions in India

**DOI:** 10.3389/fpubh.2025.1696787

**Published:** 2025-12-23

**Authors:** Anuj Kumar Pandey, Anju Pradhan Sinha, Ramu Rawat, Ranadip Chowdhury, Shivaprasad S. Goudar, Jitender Nagpal, Shrey Desai, Avula Laxmaiah, Kalpana Basany, Sadhana Joshi, Chittaranjan Yajnik, Aparna Mukherjee, Pratibha Dwarkanath, Priyanka Gupta Bansal, Molly Jacob, Shinjini Bhatnagar, Komal Shah, Debarati Mukherjee, Amlin Shukla, Raghu Pullakhandam, Varsha Dhurde, Aditi Apte, Rajeev Singh, Aakriti Gupta, Yamini Priyanka, Usha Dhingra, Ravi Prakash Upadhyay, Sutapa Bandyopadhyay Neogi, Manjunath S. Somannavar, Anirban Mandal, Gayatri Desai, Shantanu Sengupta, Shailendra Dandge, Girija Wagh, Urmila Deshmukh, Gunjan Kumar, Anura V. Kurpad, G. S. Toteja, Nikhitha Mariya John, Shailaja Sopory, Somen Saha, Giridhar R. Babu, Anandika Suryavanshi, Ravindranadh Palika, Archana Patel, Radhika Nimkar, Gaurav Raj Dwivedi, Umesh Kapil, Dilip Raja, Arup Dutta, Sunita Taneja, Diksha Gautam, Avinash Kavi, Swapnil Rawat, Kapilkumar Dave, Rajiva Raman, Catherine L. Haggerty, Sanjay Lalwani, Prachi Phadke, Alka Turuk, Tinku Thomas, Neena Bhatia, Manisha Madai Beck, Lovejeet Kaur, Aakansha Shukla, R. Deepa, Lindsey M. Locks, Dhiraj Motilal Agarwal, Raja Sriswan Mamidi, Harshpal Singh Sachdev, Rounik Talukdar, Sayan Das, Nita Bhandari, Ranjana Singh, S. Yogeshkumar, Ramasheesh Yadav, P. S. Reddy, Sanjay Gupte, S. Rasika Ladkat, Zaozianlungliu Gonmei, Swati Rathore, Dharmendra Sharma, Apurvakumar Pandya, Yamuna Ana, Patricia Hibberd, Himangi Lubree, Anwar Basha Dudekula, Priti Rishi Lal, Pearlin Amaan Khan, Aruna Verma, Umesh S. Charantimath, Indrapal I. Meshram, Karuna Randhir, Onkar Deshmukh, Ashok Kumar Roy, Obed John, Nolita Dolcy Saldanha, Ashish Bavdekar, Raj Kumar, Shyam Prakash, Wafaie W. Fawzi, Sunil Sazawal

**Affiliations:** 1 Department of Health Systems and Implementation Research, International Institute of Health Management Research, New Delhi, India; 2 Indian Council for Medical Research, New Delhi, India; 3 Centre for Public Health Kinetics, New Delhi, India; 4 Society for Applied Studies, New Delhi, India; 5 J N Medical College, KLE Academy of Higher Education and Research, Belagavi, Karnataka, India; 6 Sitaram Bhartia Institute of Science and Research New Delhi (SBISR), New Delhi, India; 7 Society for Education, Welfare and Action–Rural, Bharuch, India; 8 ICMR-National Institute of Nutrition, Hyderabad, India; 9 SHARE India, MediCiti Institute of Medical Sciences, Hyderabad, India; 10 Interactive Research School for Health Affairs, Bharati Vidyapeeth Deemed to be University, Pune, India; 11 KEM Hospital Research Centre, Pune, India; 12 St. John’s Research Institute, Bangalore, India; 13 Christian Medical College, Vellore, India; 14 Translational Health Science and Technology Institute, Faridabad, Haryana, India; 15 Indian Institute of Public Health Gandhinagar, Gandhinagar, India; 16 Public Health Foundation of India, Bangalore, India; 17 Lata Medical Research Foundation, Nagpur, India; 18 Vadu Rural Health Program, KEM Hospital Research Centre, Pune, India; 19 ICMR-Regional Medical Research Centre, Gorakhpur, India; 20 All India Institute of Medical Sciences, New Delhi, India; 21 Council of Scientific and Industrial Research-Institute of Genomics and Integrative Biology (CSIR-IGIB), New Delhi, India; 22 Bharati Hospital and Research Centre, Pune, India; 23 Department of Population Medicine, College of Medicine, QU Health, Qatar University, Doha, Qatar; 24 Banaras Hindu University (BHU), Varanasi, India; 25 School of Public Health, University of Pittsburgh, Pittsburgh, PA, United States; 26 Lady Irwin College, New Delhi, India; 27 Department of Health Sciences, Boston University, Boston, MA, United States; 28 Ahmedabad University, Ahmedabad, Gujarat, India; 29 Indian Institute of Public Health, Delhi, India; 30 Gupte Hospital and Research Centre, Pune, India; 31 Boston University School of Medicine, Corsstown, Boston, MA, United States; 32 LLRM Medical College, Meerut, Uttar Pradesh, India; 33 Avon and Wiltshire Mental Health Partnership NHS Trust, Bath, United Kingdom; 34 BRD Medical College, Gorakhpur, Uttar Pradesh, India; 35 Department of Global Health and Population, Harvard T.H. Chan School of Public Health, Boston, MA, United States

**Keywords:** anemia, iron-deficiency, intervention, public policy and governance, sustainable development goals

## Abstract

**Purpose:**

The PRAYAS Individual Patient Data Meta-analysis (IPD-MA) database aims to estimate the prevalence of anemia among children under 18 years, non-pregnant and non-lactating (NPNL) women, and pregnant women (by trimester), with further stratification by age group, year, and region of India. Beyond prevalence, it seeks to address the etiological contribution of iron and other erythropoietic micronutrient deficiencies and to evaluate the effectiveness of anemia prevention and treatment interventions, including factors associated with non-response. This will directly support India’s “test–treat–track” approach under the Anemia Mukt Bharat program.

**Participants:**

Children (0–18 years), pregnant women, and NPNL women in India.

**Findings to date:**

The database currently includes 88 datasets (1994–2023), with 319,721 participants for prevalence analysis—children (19,762), NPNL women (17,883), and pregnant women (282,076). Intervention studies comprise 59,292 participants—children (13,435), NPNL women (11,594), and pregnant women (34,263). Over half the datasets (55.7%, 49/88) are randomized controlled trials, while 35.2% (31/88) are observational. Geographically, 43.2% (38/88) are from northern India, 22.7% (20/88) from the west, and 18.2% (16/88) from the south. Most studies (67%, 59/88) are community-based. Median ages were 26 years (IQR 23–32) for NPNL and 23 years (IQR 21–25) for pregnant women, while children’s data covered 6 months to 18 years. Mean gestational age at enrollment in pregnancy was 10.24 weeks (SD 17.65). Of the total sample, 10.8% had complete blood count data, 9% ferritin, and 4.5% vitamin B12.

Among interventions, pregnant women received intravenous iron sucrose, ferric carboxymaltose, iron isomaltoside, combined IV iron with vitamin B12/folic acid/niacinamide, integrated packages, and low-dose calcium supplementation. NPNL women were often part of trials comparing 60 mg daily ferrous sulfate with 120 mg on alternate days. Children’s interventions mainly included ferrous sulfate, food supplementation, and select Ayush-based approaches.

**Future plans:**

PRAYAS will generate robust, policy-relevant evidence to refine anemia prevention and treatment strategies. Findings will directly inform the Anemia Mukt Bharat program, supporting targeted, evidence-driven interventions to reduce anemia and associated health burdens across children, women, and pregnant populations in India.

**Clinical Trial Registration:**

OSF—https://doi.org/10.17605/OSF.IO/6YRXF.

## Highlights

- The harmonized PRAYAS pooled Indian dataset is one of the largest, reliable, and most comprehensive datasets on pregnant/non-pregnant and non-lactating women and children.
- One of its kind of dataset with information on hemoglobin levels, relevant biochemical and key micronutrients parameters, and varied interventions from across India.
- Heterogeneity of interventions, dosage, duration, and data collection approaches.
- Studies lack critical parameters needed to assess changes in hemoglobin concentration like non-availability of key erythropoietic micronutrients in most of the studies, limiting the scope of certain analyses.

## Introduction

Nutritional anemia remains a significant global public health challenge (1, 2), with profound implications for the health and productivity of women and children. In 2019, anemia affected approximately one-third of women of reproductive age (WRA) globally, with approximately 269 million children aged 6–59 months also impacted (3). The burden of anemia is disproportionately high in low- and middle-income countries (LMICs) (4), with the African and South-East Asian regions contributing the most to global prevalence (5, 6). In LMIC, anemia affects 43% of the population compared to just 9% in developed nations, with WRA and children being the most vulnerable groups (7, 8). While anemia has a multifactorial etiology, iron deficiency is the most prevalent cause, particularly in LMICs, where nutritional deficiencies are widespread (9). The World Health Organization (WHO) Global Nutrition Targets aim for a 50% reduction in anemia among WRA by 2030, reflecting its prioritization in global health initiatives (10).

In India, the burden of anemia has shown alarming trends. The National Family Health Survey (NFHS-5) highlights an increase in anemia prevalence among WRA (from 53.1% in 2015–16 to 59.1% in 2019–21), pregnant women (from 50.3 to 52.6%), and children aged 6–59 months (from 58.6 to 67.1%) over the same period (11). However, the methods used for assessments and the interpretation of the results have highlighted several challenges, like the use of capillary blood, unlike gold standard methods of assessments through venous blood (12–14). Additionally, a national survey reported that ~41% of preschoolers, school-age children, and adolescents (aged 1–19 years) were anemic, with female adolescents experiencing higher prevalence rates (40%) than males (18%) (15). Anemia’s consequences are far-reaching, including fatigue, impaired cognitive and immune function, reduced productivity, and increased morbidity and mortality (16, 17). Addressing anemia remains a public health priority in India, as evidenced by initiatives like the Anemia Mukt Bharat (AMB) program, launched in 2018 to reduce anemia prevalence by three percentage points annually among children, adolescents, and WRA (17). The reduction of anemia is one of the important objectives of the POSHAN Abhiyaan launched in March 2018.

Despite these efforts, the prevalence of anemia remains unacceptably high (18, 19). Improving the understanding of anemia’s burden across demographic groups and evaluating the effectiveness of interventions are critical for guiding policy and program decisions. Complying with the targets of POSHAN Abhiyaan and National Nutrition Strategy set by the NITI Aayog, AMB strategy has been designed to reduce the prevalence of anemia.

There is a need to synthesize the evidence on anemia and analyze the progress made under AMB or reasons for its inadequate progress. In this context, it was decided to collate all existing data on anemia from studies conducted over more than four decades across diverse regions of the country by contacting the investigators. The primary goal of this initiative is to answer the questions that remain unanswered by individual studies. The results would feed into the AMB program recommendations for WRA and children. The results would also guide targeted strategies to reduce anemia in India. It is expected that by pooling the observational and interventional studies, we may investigate the etiological fractions of various causes of this recalcitrant public health problem and synthesize evidence on the effectiveness of several interventions used for prevention and treatment of anemia. By integrating data from a wide range of geographical, community, and healthcare settings, this extensive pooling of studies aims to provide a comprehensive and nuanced understanding of the underlying trends and patterns. Our approach not only enhances the depth and breadth of available evidence but also ensures a more representative and holistic perspective on the factors influencing health outcomes over time.

## Database description

### Selection of studies

Given the persistent and high burden of anemia in India despite ongoing initiatives, there was a recognized need for an in-depth understanding of the issues concerned with anemia across the country. In response, the Director General of the Indian Council for Medical Research (ICMR) commissioned an initiative in early 2023 to conduct a comprehensive assessment of the anemia burden in India. To facilitate this effort, ICMR constituted a committee of eminent experts in the field of anemia in India. The initial phase involved key preparatory activities, including the development of database search keywords, identification of principal investigators (PIs), and relevant studies across India, as well as contacting the study PIs. These activities were undertaken by the Secretariat at the ICMR.

A designated committee conducted the selection of studies under the chairperson’s guidance. To identify relevant studies, the committee adopted two well-established approaches. First, a systematic search of trial registries, and second, collaborative discussions with investigators of ongoing studies involving children and women (pregnant and NPNL) (20, 21). The process began with a database search of the Clinical Trials Registry of India (CTRI) (22). The initial search was conducted using designated keywords such as “anemia,” “prevalence,” “children,” “randomized controlled trial,” “intervention studies,” “anemia etiology,” “pregnant women,” “women of reproductive age,” and “non-pregnant women” (22).

The committee also expanded the search by examining cross-references from related studies, following up with leads provided by principal investigators (PIs) of studies included, and reaching out to additional researchers in the field. After identifying related studies, committee members contacted the PIs with requests for collaboration in early 2024. Once the PIs agreed, a data sharing agreement was prepared and signed by them, and several online meetings were held to discuss the details of the proposed database and the datasets involved. After the online meetings with study PIs, the harmonization meeting was held at ICMR headquarters in New Delhi in early August 2024 with the objective of discussing methodological issues, explaining to the participants what the database would be, and discussing the difficulties in filling the data extraction sheets. Such meetings were scheduled at the ICMR headquarters at routine intervals of 3–4 months of time. The meeting also aimed at handholding the PIs on how to fill in the data cells. A second round of data harmonization meeting was held at the end of October 2024. After identifying and removing duplicates, a total of 88 datasets from studies, conducted by 23 organizations at different time points in India, were included in the database synthesis following certain inclusion criteria -

- This database included studies on anemia conducted on children under 18 years, NPNL women, and pregnant women, where data on hemoglobin levels were available.
- Eligible studies included cross-sectional and interventional designs (both randomized and non-randomized), longitudinal studies, unpublished studies, including the COVID registries with due approval from the relevant authorities.
- Studies conducted in India, with data on hemoglobin and other relevant biochemical parameters (e.g., serum ferritin levels, complete blood counts, vitamin B12 levels, and inflammatory markers) at baseline and post-intervention were also included within the database.

Data sets were excluded if data on hemoglobin and other relevant biochemical parameters were not available.

### Data sources in the pooled data set

**Figure 1:**
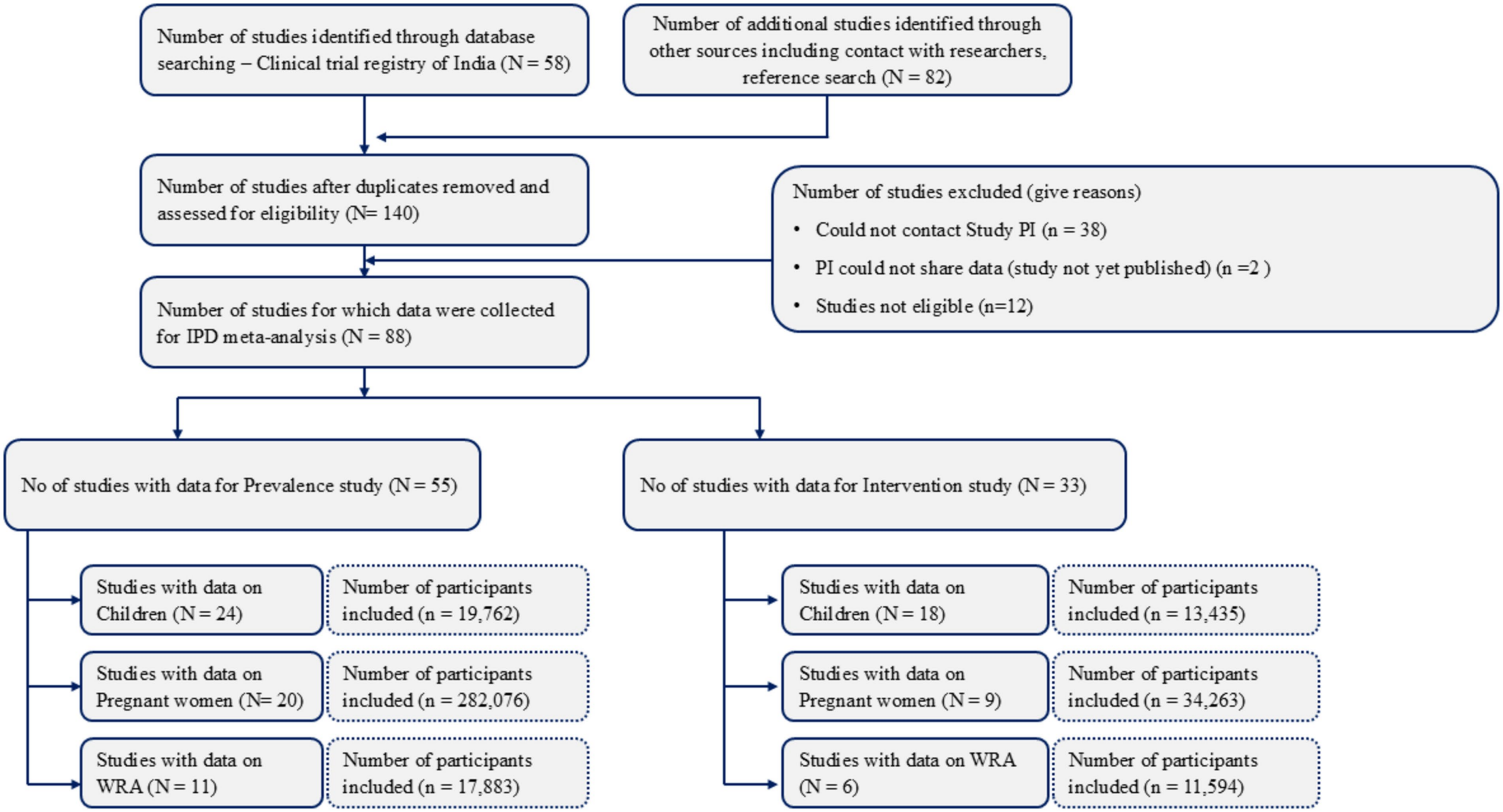
Overview of the data included in the pooled database.

After completing the formalities and harmonization, the PIs shared their anonymized datasets in a predefined format. The data included information such as study ID, woman/subject ID, demographic details, interventional strategies, hemoglobin and ferritin levels, relevant biochemical parameters at baseline and post-intervention, comorbidities, adverse effects, etc. Figure 1 provides details of the studies identified through databases and the number of studies included in the final database. The details of each of the studies included are available elsewhere (Supplementary Table 1).

The ICMR team ensured that the included studies complied with relevant ethical guidelines and regulations. All included primary studies had received approval from ethics committees recognized by the Department of Health Research in India, and informed consent was obtained from all study participants. Table 1 provides details of studies included within the database of PRAYAS.

**Table 1:** Details of studies—database profile PRAYAS.

Name of institute (no of dataset)	Sample size	Period (year of blood collection during the study)	Study setting (Community-based or hospital-based)	Region (North, West, South, Central, North-East)	Study design (observational or RCT)
Prevalence-WRA					
IIHMR—New Delhi (2)	2,457	2014–14	Hospital	North, South, East	Observational
	838	2018–19	Hospital	South, East	Observational
KEM Hospital, Pune (2)	691	2001–03	Community	West	Observational
	656	2006–08	Community	West	Observational
SAS—New Delhi (3)	408	2018–2019	Community	North	RCT
	907	2017–2021	Community	North	RCT
	6,672	2017–2021	Community	North	RCT
ICMR-NIN, Hyderabad (1)	470	2019	Hospital	South	Longitudinal
ICMR-RMRC, Gorakhpur (1)	536	2022–23	Community	North	Observational
ICMR—Headquarter (1)	4,128	2020–22	Hospital	Across India	Observational
CMC Vellore (1)	120	2021	Hospital	South	Longitudinal
Total sample	17,883				
Intervention-WRA					
SAS—New Delhi (4)	816	2018–2019	Community	North	RCT
	4,069	2017–2021	Community	North	RCT
	4,069	2017–2021	Community	North	RCT
	2,050	2017–2021	Community	North	RCT
CMC, Vellore (1)	120	2020–21	Hospital	South	RCT
ICMR-NIN, Hyderabad (1)	470	2019	Community	South	Longitudinal
Total sample	11,594				
Prevalence—pregnant					
CMC—Vellore (3)	107	2019–22	Hospital	South	Longitudinal
	107	2019–22	Hospital	South	Longitudinal
	107	2019–23	Hospital	South	Longitudinal
KLE (JN Medical College) Belagavi (2)	11,220	2002–23	Community	South, North	RCT
	125,180	2010–19	Community	South	Observational
IIPH—Bengaluru (2)	1,634	2016–19	Hospital	South	Observational
	1,317	2016–19	Hospital	South	Observational
KEM Hospital—Pune (2)	737	1994–95	Community	West	Observational
	670	1994–95	Community	West	Observational
SAS—New Delhi (2)	2,269	2017–2021	Community	North	RCT
	910	2017–2021	Community	North	RCT
SEWA Rural—Gujarat (1)	458	2023	Hospital	West	Observational
ICMR—Headquarter (1)	16,539	2021	Hospital	Across India	Observational
ICMR—Headquarter (1)	874	2020–22	Hospital	Across India	Observational
THSTI—Faridabad (1)	6,000	2015–23	Hospital	North	Observational
	8,665				
	6,481				
IIPH—Gandhinagar (1)	207	2020	Community	West	Observational
MIMS—Telangana (1)	1,257	2010–18	Hospital	South	Longitudinal
Bharati Vidyapeeth, Pune (1)	1,062	2017–21	Hospital	West	Observational
Lata Foundation Nagpur (1)	85,277	2010–2021	Community	Central	Observational
SJRI Bangalore (1)	10,998	2018–22	Hospital	South	RCT
Total sample	282,076				
Intervention-pregnant					
SAS—New Delhi (3)	4,081	2017–21	Community	North	RCT
	4,081	2017–21	Community	North	RCT
	2,059	2017–21	Community	North	RCT
KLE (JN Medical College) Belagavi (2)	2,912	2022–23	Community	South, North	RCT
	2,906	2022–23	Community	South, North	RCT
IIHMR—New Delhi (1)	1,999	2017	Hospital	North, East	RCT
SJRI—Bengaluru (1)	10,998	2018–22	Hospital	South	RCT
SEWA Rural—Gujarat (1)	100	2017–18	Hospital	West	Pre-post
MIMS Hyderabad, Telangana	5,127	2009–18	Hospital	South	Observational Cohort
Total sample	34,263				
Prevalence children					
SAS—New Delhi (5)	517	2018–2019	Community	North	RCT
	408	2018–2019	Community	North	RCT
	652	2021–2022	Community	North	RCT
	1,300	2021–2022	Community	North	RCT
	319	2020–2021	Community	North	RCT
CPHK—New Delhi (4)	1,257	2002–2004	Community	North	RCT
	3,002	2014–15	Community	North	RCT
	300	2009–2011	Community	North	RCT
	2,250	2017–19	Hospital	North	RCT
KEM Hospital, Pune (3)	704	2001–03	Community	West	Observational
	685	2005–08	Community	West	Observational
	685	2012	Community	West	Observational
KEM Vadu—Pune (2)	972	2004	Community	West	RCT
	551	2007	Community	West	RCT
SBISR—New Delhi (1)	100	1999	Hospital	North	RCT
ICMR—RMRC-Gorakhpur (1)	1,017	2022–23	Community	North	Observational
IIPH—Bengaluru (1)	256	2023–24	Hospital	South	Observational
IIPH, Gandhi Nagar (1)	450	2021	Community	West	RCT
Lata Foundation—Nagpur (1)	225	2020	Community	Central	Observational
AIIMS, New Delhi (1)	1,054	2017	Community	North	Observational
ICMR—Headquarter (1)	658	2020–22	Hospital	Across India	Observational
ICMR—Headquarter (1)	446	2015	Community	North	Observational
ICMR—Headquarter (1)	446	2020–22	Hospital	Across India	Observational
NIN—Telangana (1)	1,508	2017	Community	Central, Northeast, South, West, East	Observational
Total sample	19,762				
Intervention children					
SAS—New Delhi (8)	816	2018–2019	Community	North	RCT
	1,036	2018–2019	Community	North	RCT
	1,029	2018–2019	Community	North	RCT
	1,300	2021–2022	Community	North	RCT
	1,300	2021–2022	Community	North	RCT
	1,678	2020–2021	Community	North	RCT
	1,678	2020–2021	Community	North	RCT
	837	2020–2021	Community	North	RCT
KEM Vadu—Pune (5)	184	2004–05	Community	West	RCT
	167	2004–05	Community	West	RCT
	165	2004–05	Community	West	RCT
	165	2004–05	Community	West	RCT
	414	2007	Community	West	RCT
IIPH—Gandhi Nagar (1)	245	2022	Community	West	RCT
MIMS—Telangana (1)	1,286	2010–18	Hospital	South	Observational
ICMR—Gorakhpur (1)	461	2023	Community	North	RCT
SBISR—New Delhi (1)	100	1999–2000	Hospital	North	RCT
AIIMS, New Delhi (1)	1,054	2017	Community	North	RCT
Total sample	13,435				

After finalizing the datasets, the entire database was separated into groups for children under 18 years, NPNL, and pregnant women. The studies were then categorized into two groups:

1. Prevalence studies and
2. Intervention studies

This categorization was important since it dictated the type of analysis and statistical methods to be applied. The included studies (Table 1) span both hospital and community settings and include observational, longitudinal, randomized controlled trials (RCTs), and pre–post-intervention designs. States and UTs were classified as regions for analysis following the classification system set by the Registrar General & Census Commissioner of India for sample registration system (SRS) (23).

### Variable availability and definition

Data harmonization is a critical step while developing a database profile, ensuring that data from diverse studies can be integrated and analyzed collectively, thus enhancing the reliability and generalizability of the findings (24–26). A significant aspect of harmonization was to ensure uniform units for all biochemical variables. For example, hemoglobin levels (reported in grams per deciliter or grams per liter by different studies) were standardized to a single unit (grams per deciliter). This step, along with the standardization of other blood parameters such as red blood cell count and serum ferritin, was also undertaken. A separate sheet with standardized parameters was developed for reference (Table 2).

**Table 2:** Cutoff values for hemoglobin along with the unit for data collection.

Hemoglobin cutoff	Unit	Children 6–23 months of age	Children 6–59 months of age	NPNL	Pregnant women (first and third trimester)	Pregnant women (second trimester)
Non-anemia	gm/dl	10.5 or higher	11.0 or higher	12.0 or higher	11.0 or higher	10.5 or higher
Mild anemia		9.5–10.4	10.0–10.9	11.0–11.9	10.0–10.9	9.5–10.4
Moderate anemia		7–9.4	7.0–9.9	8.0–10.9	7.0–9.9	7–9.4
Severe anemia		<7.0	<7.0	<8.0	<7.0	<7.0

Hemoglobin (Hb), the primary outcome indicator of anemia, was measured in grams per deciliter (g/dL), and categorized as mild, moderate, and severe based on the hemoglobin threshold as mentioned in the updated guideline on hemoglobin cutoffs to define anemia, released in 2024 (Table 2) (27, 28).

Table 3 also presents the acceptable upper and lower values for each hematological and biochemical biomarkers for children, pregnant, and non-pregnant women. These values served as quality control measures to exclude implausible values. Additionally, the table also presents the acceptable unit for each parameter. Definition for the micronutrient-related thresholds, inflammatory, and metabolic markers was also defined to check for the quality of collected data.

**Table 3:** Acceptable values to eliminate abnormal values from the database.

Parameter	Children		Pregnant women		Non-pregnant women	
	Lower acceptable value	Upper acceptable value	Lower acceptable value	Upper acceptable value	Lower acceptable value	Upper acceptable value
Hematocrit (36)	<30%	>44.1%	<36%	>48%	<36%	>48%
MCV (37)		>86 femtoliter (fL)	<80 femtoliter (fL)	>100 femtoliter (fL)	<80 femtoliter (fL)	>100 femtoliter (fL)
MCH (38, 39)	(6 m–1 yr)23 pg	(6 m–1 yr) 31 pg	>33 picograms (pg) per cell		>33 picograms (pg) per cell	
MCHC (40)	(6 m–1 yr) < 32 g/dL	(6 m–1 yr) > 36 g/dL	<32 g/dL	>36 g/dL	<32 g/dL	>36 g/dL
Ferritin (41, 42)		140 μg/L	13 μg/L	150 μg/L	13 μg/L	150 μg/L
Transferrin saturation (43–45).	(0 to <1 year) 4.1%	30% (0 to <1 year) 59%	15%	50%	15%	50%
sTfR (46).				4.4 mg/L		4.4 mg/L
μg/dL (46, 47).		(Abnormal values) 3–6 years >70 μmol/mol heme		100 μg/㎗		100 μg/㎗
Vitamin A (48, 49)	<0.70 μmol/L		0.07 μmol/g	3,000 retinol activity equivalents (RAE)/Day	0.07 μmol/L	3,000 retinol activity equivalents (RAE)/Day
Vitamin B12 (50, 51)	<150 pmol/L (203 pg./mL)		100 pmol/L	350 pmol/L	100 pmol/L	350 pmol/L
Folate (Serum) (52, 53)	<4 ng/mL (<10 nmol/L)		2.0 ng/mL	7.0 ng/mL	2.0 ng/mL	7.0 ng/mL
Folate (RBC) (53, 54)	<151 ng/mL (<340 nmol/L)			>400 ng/mL		>400 ng/mL
Zinc (55)	<10 years:65 mg/dL		<10 years:65 mg/dL	70 mcg/dL	<56 (μg/dL)	70 mcg/dL
Vitamin D (56–58)	<12 ng/mL		<30 nmol/L		10 ng/mL	50 ng/mL
CRP (59)	> 5 mg/L		0.1 mg/L	>5.0 mg/L	0.1 mg/L	>5.0 mg/L
AGP (60)	>1 g/L		0.4 mg/mL	3 mg/mL	0.4 mg/mL	3 mg/mL
IL-6 (55)			5 pg./mL	25 pg./mL	5 pg./mL	25 pg./mL
D-Dimer (61)			500 ng/mL	10,000 ng/mL	500 ng/mL	10,000 ng/mL

RDW, red cell distribution width; MCV, mean corpuscular volume; MCH, mean corpuscular hemoglobin; MCHC, mean corpuscular hemoglobin concentration; sTfR, soluble transferrin receptor; ZnPP, zinc protoporphyrin; CRP, C-reactive protein; AGP, alpha-1-acid glycoprotein; IL-6, interleukin-6.

### Principles and plans for statistical analysis

A detailed statistical analysis and reporting plan was formulated in collaboration with the Technical Advisory Group of the PRAYAS consortium. This plan delineated the statistical techniques, underlying assumptions, and procedural steps, ensuring systematic, and transparent analyses (details will be reported in subsequent papers). One-stage meta-analysis and two-stage meta-analysis approaches would be used for analyzing all the available data to calculate the prevalence of anemia and its severity across age groups. Using a weighted sample, the prevalence of anemia and its severity will be calculated as the number of anemics divided by the total number of participants in different age groups. To account for differences in sample sizes across datasets, each dataset will be weighted, with *weights computed as the inverse of the ratio of the individual dataset sample size to the overall pooled database sample size*.

The analysis will use a one-stage individual participant data meta-analysis approach, pooling harmonized data from all included studies to estimate the adjusted etiological fractions of anemia due to specific micronutrient deficiencies (such as iron, folate, vitamin B12, vitamin A, vitamin D, and zinc) in children, non-pregnant/non-lactating women, and pregnant women (by trimester where possible). Multilevel regression models will be employed, with study as a random effect and relevant covariates included to account for confounding and between-study heterogeneity. Adjusted risk ratios for each deficiency will be used to calculate PAFs, with subgroup analyses by age, region, and other modifiers. Sensitivity analyses will assess the robustness of findings to different deficiency cut-offs and model specifications. To evaluate intervention effects, logistic regression will estimate relative risks (RR) for anemia prevalence, while linear regression will compute mean differences (MD) in hemoglobin levels, adjusted for confounders. Additionally, the database would also be utilized to develop risk prediction models using machine learning approaches.

### Patient and public involvement

No patients or members of the public were directly involved in the design or conduct.

## Findings to date

The PRAYAS database, spanning over 379,013 individuals, offers a rich, regionally diverse, and methodologically varied resource to derive meaningful insights into nutritional anemia across India.

### Study profile

Pooled database comprises 88 datasets, encompassing a total of 319,721 participants for prevalence analysis—children (19,762), NPNL (17,883), and pregnant women (282,076). Additionally, 59,292 participants were included in intervention studies—children (13,435), NPNL (11,594), and pregnant women (34,263). RCTs comprised 55.7% (49/88) of the datasets, whereas observational studies comprised 35.2% (31/88) of the datasets. Others were longitudinal studies (8%–7/88) and pre–post-study (1.1%–1/88). The included studies were conducted across various regions of India: the major contributions were from the northern region of India with 38 studies (43.2%), followed by the western part of India with 20 studies (22.7%). The southern part contributed 16 studies (18.2%). A smaller share from the central part of India (2.3%–2/88) followed by 12 studies (13.6%) from across India or have spanned multiple regions. These studies span from 1994 to 2023. A majority [59/88 (67%)] of the datasets originates from community-based studies, while 29/88 (33%) were derived from hospital-based research (Table 1).

### Baseline characteristics—prevalence datasets

Of the included studies, more than 85% of the sample had information on hemoglobin concentration with highest among the pregnant women datasets with information from 96.1% (270,939) sample followed by NPNL and children with 94.4% (16,878) and 87.8% (17,351), respectively. The sample included NPNL and pregnant women with a median age of 26 years (IQR 23–32) and 23 years (IQR 21–25), respectively. Within the children datasets, information from 6 months up to 18 years was pooled within the database. Ultrasonography was used in 76.8% (198,819) of the sample for gestational age assessment (23.2%–60,053 used the LMP method). The mean gestational age at enrollment was 10.24 weeks (SD*—*17.65). Specifically, more than one-third (41.32%–105,103) of the participants were enrolled in the first trimester of pregnancy, whereas 37% (94,249) in the second trimester.

**Figure 2:**
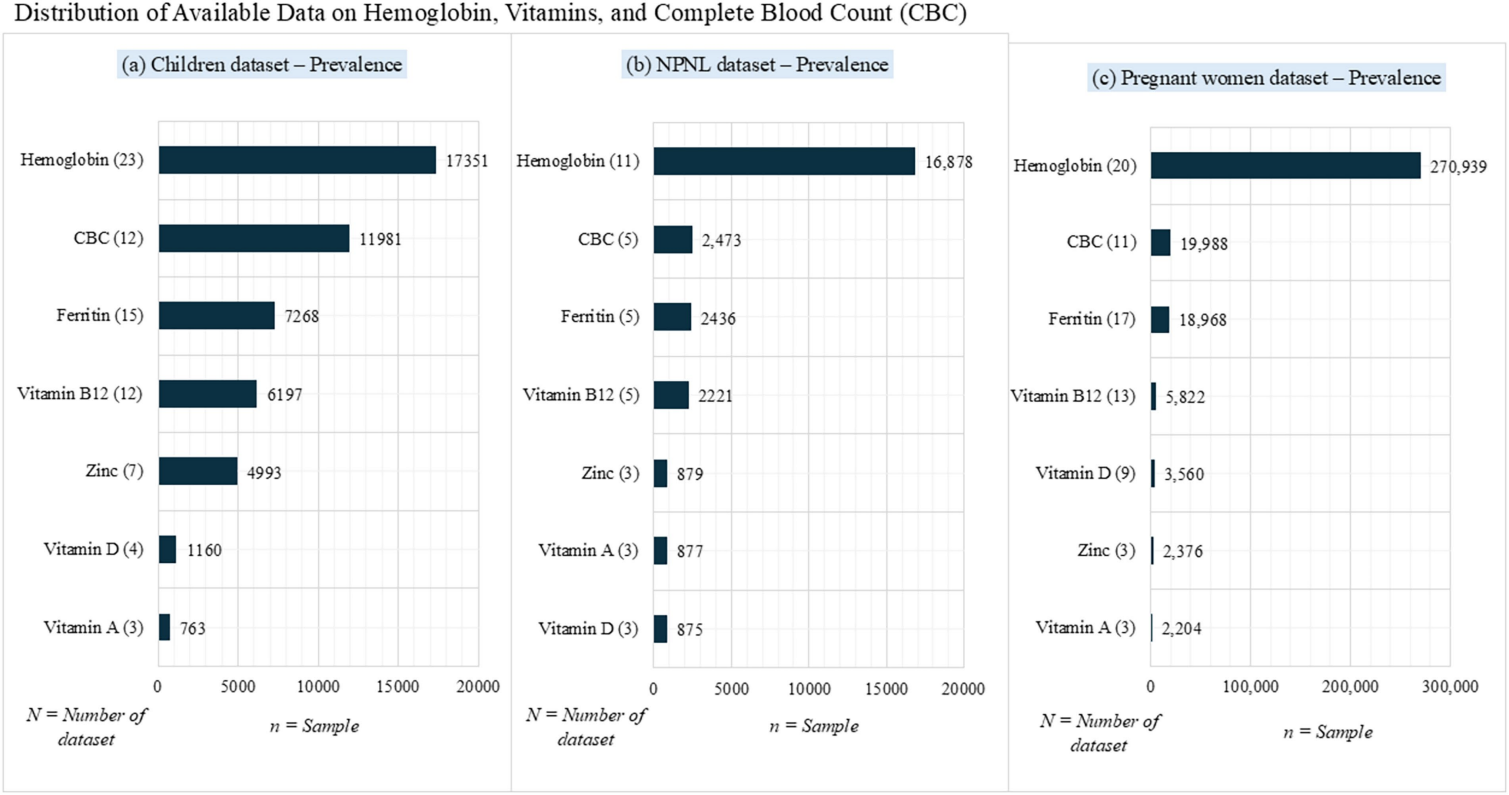
Distribution of available data on hemoglobin, vitamins, and complete blood count (CBC): **(a)** Children dataset; **(b)** NPNL dataset; **(c)** Pregnant women dataset.

Further assessment of information on each hematological and biochemical biomarker reported that overall, 10.8% (34,442/319,721) of the sample had information on complete blood count (CBC). Whereas of the total sample, 9% (28,672) and 4.5% (14,240) had information on ferritin and vitamin B12, respectively. Less than 5% of the sample had information on other essential parameters (Figure 2). We are yet to analyze other essential parameters within the database. Figure 2 illustrates the distribution of available data on hemoglobin, vitamins, and complete blood count (CBC) among children, NPNL, and pregnant women dataset.

### Baseline characteristics—intervention datasets

Within the PRAYAS database, a total of 33 datasets (sample*—*59,292) were from intervention studies. Of these, 87.9% (29/33) datasets are randomized controlled trials with maximum within the children database (17 datasets). This dataset focuses on addressing anemia through nutritional and therapeutic approaches.

For the pregnancy database, 23.8% (8,150/34,263) of the interventions were specified as therapeutic. It is pertinent to note that 54.6% (18,719) of the samples were not specified within the one single category of therapeutic or preventive. Among pregnant women, a broader range of interventions was implemented, including intravenous iron sucrose (18), ferric carboxymaltose (IV FCM) (29), iron isomaltoside (IV IIM) (29), IV iron combined with vitamin B12, folic acid, and niacinamide, integrated interventions (a combination of health, nutrition, psychosocial care, and WASH) (30, 31), as well as low-dose calcium supplementation (32). These were administered either during pregnancy alone, during both preconception and pregnancy, or in the preconception period only (30, 31). The control groups primarily received either high-dose calcium in one study (32) or oral iron in the rest others.

For the WRA group, 53.9% (6,246/11,594) of interventions were categorized as therapeutic. A total of 4 study namely WINGS (30, 31), IMPRINT (33), ICMR NIN study (34), CMC-RCT contributed to the database. WINGS provided integrated interventions (a combination of health, nutrition, psychosocial care, and WASH). These interventions were delivered at different stages, namely during preconception, during preconception + pregnancy, and during pregnancy with a control of oral iron. A study by CMC compared ferrous sulfate tablets of 60 mg elemental iron daily with a control of 120 mg on alternate days. Whereas NIN study administered prophylactic IFA and assessed for iron deficiency anemia in pre–post-method. Lastly, IMPRINT study provided food supplements and compared them with the oral iron group.

Within the children’s datasets, 7 studies contributed to a total of 18 datasets (sample—13,435). First study IMPRINT (33) contributed to a total of eight dataset delivered interventions as supplement or food vehicle, whereas others have delivered interventions as supplement or through fortification. Studies have administered ferrous sulfate as interventions along with food supplements, and some were Ayush trials.

## Discussion

The PRAYAS database is a compilation of datasets from India on Anemia among women and children. This compilation is in response to prolonged deliberations regarding stagnancy in the prevalence of anemia in India despite focused interventions like AMB. Studies have explained an increase in compliance with such programmatic interventions that can accelerate reductions in anemia prevalence (35). Despite such decisive interventions and framework, findings from nationally representative sample surveys highlight an increase in anemia prevalence among WRA (from 53.1% in 2015–16 to 59.1% in 2019–21), pregnant women (from 50.3 to 52.6%), and children aged 6–59 months (from 58.6 to 67.1%) over the same period in India (11). Another study noted that there is an obvious shift in the distribution of Hb to the right among pregnant women over the past several years (28). This shift could be attributed to the implementation of the programmatic interventions with a focus on pregnant women or to factors stemming from overall development. The dearth of robust evidence around the diverse clinical etiologies of anemia, effective interventions, etc., demands a study that can be used for further policy decision-making.

### Strengths and limitations

Data synthesized from the pooled data database would be used for calculating anemia indicators for the given population as these data have been collected from high-quality and closely observed observational and randomized controlled studies mostly using venous blood samples. This is an important resource considering several challenges associated with the existing health surveys (12–14). Additionally, the analysis would provide etiological fractions for anemia prevalence importantly fraction due to iron deficiency in all age groups of children under 18 years, NPNL, and pregnant women. These details can help the program to decide on the necessity of continuing prophylactic supplementation for these age groups and also finetune the doses for the same. Furthermore, the individual patient data meta-analysis of intervention studies can inform robust evidence regarding the type of iron intervention and dose of iron in both therapeutic and prophylactic studies. Additional social parameters could further enrich the analysis; these were not included due to the nature of the secondary data used.

The analysis from this database is expected to generate robust, high-quality evidence from large high-quality studies to inform public health policies and guide strategies for reducing the anemia’s burden in India. The systematic harmonization approach employed in this study ensures the validity and reliability of the datasets by addressing variations in data collection and standardizing outcome measures. This methodological rigor will enable more precise estimates and facilitate meaningful comparisons across populations and interventions (24–26).

However, several limitations should be noted. First, pooling data from studies with varying intervention types may result in high heterogeneity, which will be addressed through subgroup and sensitivity analyses. Second, some studies may lack critical parameters needed to assess changes in hemoglobin concentration, limiting the scope of certain analyses. Additionally, challenges in obtaining participant-level data due to restrictions from principal investigators or unpublished results could lead to data gaps. The inclusion of heterogeneous intervention and control conditions may also introduce a risk of bias, complicating the generalizability of findings. To mitigate these issues, we will evaluate heterogeneity using advanced statistical models, such as random-effects meta-analysis, and conduct subgroup analyses to explore the impact of differences across geographic, demographic, and intervention-specific factors.

The ability to analyze participant-level data allows for greater flexibility in adjusting for confounders, exploring effect modifiers, and conducting tailored subgroup analyses. By addressing sources of heterogeneity and potential biases, this meta-analysis aims to provide nuanced and reliable insights into the epidemiology of anemia and the effectiveness of various interventions.

Analyses from this database, to be presented in subsequent manuscripts, will provide findings that enhance understanding of the factors driving the high prevalence of anemia in India and the effectiveness of interventions to address this public health challenge. The findings will support evidence-based policymaking, i.e., will feed into the Anemia Mukt Bharat program recommendations for WRA and children and guide targeted strategies to reduce anemia and its associated health burdens across vulnerable populations.

## Data Availability

The data analyzed in this study are subject to the following licenses/restrictions: All the collaborating PIs have acknowledged that the pooled data can only be used for this IPD analysis, with no transfer of ownership. Requests to access these datasets should be directed to apradhandr@gmail.com.

## Author’s note

Study findings will be published in peer-reviewed journals and will also be communicated to the policy makers for effective decision-making to curb the increasing trend of anemia in India. Commissioned by the Indian Council of Medical Research—India.
