# Supplementary material for: PRAYAS: individual patient data meta-analysis database for Pooled Research and Analysis for Yielding Anemia-free Solutions in India
Source: Front Public Health. 2025 Dec 23;13:1696787. doi: 10.3389/fpubh.2025.1696787 (PMC12771771; doi:10.3389/fpubh.2025.1696787)
Supplement: Supplementary file 1 [file Table_1.docx]

**Supplementary File**

**PRAYAS: Cohort profile for Pooled Research and Analysis for Yielding Anemia-free Solutions in India**

[S Table 1: Summary table for pooled data analysis 2](#_Toc204522856)

# S Table 1: Summary table for pooled data analysis

| **Sr No** | **Study name** | **Type of data ( children/ pregnant/ NPNL)** | **Assessment details** | **Study outcome** | **Study Reference** |
| --- | --- | --- | --- | --- | --- |
| **1** | Sprinkles | Children (6-18 months of age) | Hemoglobin, ferritin | Change in hemoglobin and ferritin | Hirve S et.al (1) |
| **2** | Iron Atta | Children (6-15 years) | Hb, serum ferritin, transferrin receptor, zinc protoporphyrin | Prevalence of anemia and iron deficiency anemia | Muthayya S et.al (2) |
| **3.** | FeSPAW | Pregnant women | Maternal and fetal outcome, Hemoglobin, ferritin | Maternal outcome, fetal outcome, and change in hemoglobin and ferritin | Neogi et.al (3) |
| **4** | HTA2015 | For the present study – non-pregnant women aged 18-88 years | Hemoglobin using autoanalyzer | Hemoglobin | Neogi et.al (4) |
| **5** | HTA2019 | For the present study – non-pregnant women aged 18-60 years | Hemoglobin using autoanalyzer | Hemoglobin | Neogi et.al (5) |
| **6** | LIFE | Pregnant women | Hemoglobin | Prevalence of anemia ,Change in Hb levels in pregnancy | Kusneniwar GN et al (6) |
| **7** | LIFE | Child | Hemoglobin | Preterm births and anemia |  |
| **8** | Iron Supplementation in Breastfed infants. | Infant | Hemoglobin and ferritin at enrollment, 4 weeks and 8 weeks | Change in Hemoglobin | Nagpal J et.al (7) |
| **9** | GARBH-Ini | Pregnant women | Hemoglobin on most.  A subset of 2000  participants at < 20 weeks has:  Zinc, Vitamin D Vitamin B12, Folate, Iron, Transferrin saturation, Ferritin, CRP | Preterm Birth | Thiruvengadam R et.al (8)  Bhatnagar S et.al (9) |
| **10** | PMNS | Pregnant women | Hemoglobin, Ferritin, Vitamin_b12, Folate_rbc. | 1. Maternal determinants of fetal growth, and 2. Life-course evolution of offspring phenotype (NCD) | Yajnik C S et.al (10,11)  Rao S et.al (12) |
| **11** |  | Women | Hemoglobin, Vitamin_b12, Folate serum. |  |  |
| **12** |  | Children | Hemoglobin, ferritin, Vitamin_b12, folate serum, CRP |  |  |
| **13** | MAASTHI | Pregnant | Hb ( ~ 2951 PW), B12, Vitamin D, folate, sTfR, ferritin, CRP (~ for 231 PW) | Child weight, adiposity | Babu GR et al. (13–15)  Lobo Eunice et.al (16) |
|  |  | Children (3 to 7 years) | CBC, Haemoglobin | Cognition and mental health | Lobo Eunice et.al (17) |
| **14** | Estimation of prevalence of anemia and coverage of adolescent anemia control programs among girls aged 15-19 years in Nagpur District, India | 14-19 years adolescent girls | Hb, CBC, ferritin, iron, transferrin, TIBC, saturation, folate, B12, CRP | Anemia prevalence, micronutrient deficiency, coverage of anemia control program | Dhurde V S et.al (18) |
| **15** | RAJGKP2022 | Children from 6 months (mths.) to below 60 mths. | Hb, CBC, ferritin,  folate, B12, Vit-D, CRP, Anthropometric data | Prevalence of anemia and iron deficiency anemia | Not published |
| **16** | RAJGKP2022 | Women of reproductive age | Hb and CBC | Prevalence of anemia | Not published |
| **17** | RAJGKP2022 | Children from 6 mths to below 60 mths | Education of mothers of anemic children by lay health workers and its effect on IFA therapy | Education improved in recovery from anemia by IFA therapy | Not published |
| **18** | MNHR | Pregnant woman | Hb, Anthropometry (Height and Weight),, SES, Maternal and Neonatal mortality and morbidity outcomes through 42 days postpartum | Population trends over time – Maternal and Neonatal Mortality and Morbidity, Early pregnancy loss, Still births, Low birth weight,  Preterm  births, Maternal Anemia | Goudar S S et.al (19) |
| **19** | RAPIDIRON | Pregnant woman | Hb, Ferritin, TSAT, Vitamin B12, Serum folate, CBC, Retic Hb and IRF, Anthropometry, SES | 1.Conversion to non-anemic status by the delivery  2.Prevalence of Low birth weight | Derman RJ et al. (20) |
| **20** | WINGS | Non pregnant (18-30 years)  Pregnant women  Children-24 months | Hemoglobin  Vitamin A, D, B12, Folate, ferritin, CRP | LBW, SGA, Preterm,  Stunting, LAZ | Taneja S et.al (21) |
| **21** | Calpreg | Pregnant women | Hemoglobin, anthropometry, SES, calcium supplement. | HDP & PE and preterm as primary outcomes. | Dwarkanath et.al (22) |
| **22** | IMPRINT 1 | Children-6m | Hemoglobin  Vitamin A, D, B12, Folate, ferritin, CRP | Stunting | Taneja S et.a l(23) |
| **23** | FCM | Pregnant women | Hb | Improvement in Hb | Maitri V et a l(24) |
| **24** | Causes of Anemia among pregnancies | Pregnant women | Vitamin B12, Folate, Iron, Total Iron binding capacity, Transferrin saturation, Ferritin, CRP, haemoglobinopathies, creatinine | Cause of anemia | Not published |
| **25** | IMPRINT 2 | Children-12m | Hemoglobin  Vitamin A, D, B12, Folate, ferritin, CRP | Stunting | Taneja S et.al (25) |
| **26** | Anemia-Treatment | Children 6-59 m | Hemoglobin  Vitamin A, D, B12, Folate, ferritin, CRP | Hb, Anemia |  |
| **27** | Anemia-Prevention | Children 6-59 m | Hemoglobin  Vitamin A, D, B12, Folate, ferritin, CRP | Hb, Anemia |  |
| **28** | REVAMP | Pregnant women | Hemoglobin,  CBC | Pre-eclampsia,  GDM,  Pre-term,  Low birth weight | Wadhwani et al. (26) |
| **29** | B12 study, (including iron and folic acid study) - DBT funded | 1. 766 Adolescent boys (12-19 years)  2. 740 adolescent girls (12-19y)  3.1058 NPNL women  4. 839 Adult women (20-59y)  5. 740 Elderly women (60y and above)  6. 828 adult men (20-59y)  7.720 Elderly men (60y and above) | 1. SES/Wealth index  2. Anthropometry (Ht, wt, WC -adults)  3. Biochemical - Vit B12, folate, homocysteine, Holo-TC, CBC -Hb, Ferritin, CRP,  4. FBG -Adults and elderly  BP - adults and elderly | Prevalence of anemia, iron deficiency anemia,  B12, iron and/or folic acid anemia  and predictors of B12, folic acid and iron deficiencies | Ghosh , A. Laxmaiah, G. et al (27) |
| **30** | Etiology of anemia and B12 study | Adolescent girls (12-19 years) | Hb, B12, folate, sTfR, ferritin, CRP, Hepcidin | Impact of B12 on hb and ferritin.  Etiology of anemia | Gupta A et. al (28–30) |
| **31** | Ayurvedic intervention to improve the nutritional status under 5 children | Adolescent girls (11-18) | Hb | Impact of ayurvedic intervention on the nutritional status of adolescent girls | Jimeet Soni et.al (31) |
| **32** | Ayurvedic intervention to improve the nutritional status among adolescent girls and under 5 children at Devbhoomi Dwarka district, Gujarat | Adolescent girls (11-18) | Hb | Impact of ayurvedic intervention on the nutritional status of adolescent girls |  |
| **33** | Non-iron Deficiency Anemia in Rural Indian Women: A Cross-Sectional Study | Pregnant Women | hemoglobin and ferritin | Non-iron Deficiency Anemia in Rural Indian Women | Saha S et. al (32) |
| **34** | Efficacy of iron-folic acid treatment for reducing anemia prevalence  and improving iron status in women of reproductive age: A one-year  longitudinal study | WRA | CBP, Ferritin, CRP, sTfR | The ‘screen and treat with IFA’ approach is efficacious in reducing the prevalence of anemia  in general among WRA, with persistent and significant effects after 9 months of follow-up. | Palika R et.al (33) |
| **35** | Anemia and micronutrient status among adolescent girls residing in Kirti Nagar slums of Delhi | Adolescent girls (10-19 years) | Hb, Ferritin, Folic Acid, Vitamin B12 | Status of anemia and micronutrient deficiency among adolescent girls | Bansal PK et al (34) |
| **36** | National Registry on COVID-19 Infection among pregnant women and their neonates | Pregnant women | Hb, hct, Ferritin, |  | Not published |
|  | National Registry on COVID-19 Infection among pregnant women and their neonates | Neonates | Hb, hct, Ferritin, CRP |  | Not published |
| **37** | Prebiotic and probiotic fortified milk in prevention of morbidities among children: community-based, randomized, double-blind, controlled trial | Children | Hb,hct, rdw, mcv, mchc, mpv Ferritin, sftr, znpp, zn | Milk can be a good medium for delivery of prebiotic and probiotic and resulted in significant reduction of dysentery, respiratory morbidity and febrile illness. Overall, impact of diarrhea was not significant | Sazawal S et al (35) |
| **38** | Effects of Bifidobacterium lactis HN019 and prebiotic oligosaccharide added to milk on iron status, anemia, and growth among children 1 to 4 years old | Children | Probiotic and Prebiotic fortified milk | Preschoolers are usually fed milk, which has good acceptance and can be easily fortified for delivery of probiotics. Consumption of B lactis HN019 and prebiotic-fortified milk resulted in a smaller number of iron-deficient preschoolers and increased weight gain | Sazawal S et al (36) |
| **39** | Efficacy of high zinc biofortified wheat in improvement of micronutrient status, and prevention of morbidity among preschool children and women - a double masked, randomized, controlled trial | Children | Hb,hct, rdw, mcv, zn | Biofortified wheat flour had a good compliance among children and WCBAs. Significant improvement on some of the self-reported morbidity indicators suggests that evaluating longer-term effects of biofortification with higher grain zinc content would be more appropriate | Sazawal S et al (37) |
| **40** | Effect of dose reduction of supplemental zinc for childhood diarrhoea: study protocol for a double-masked, randomised controlled trial in India and Tanzania | Children | Hb,hct, rdw, mcv, mchc, mpv Ferritin, sftr, znpp, zn | The results of the trial are likely to be generalisable to childhood acute diarrhoea in similar resource-limited settings and may influence global policy about zinc supplementation dosage during acute diarrhea | Somji SS et al (38) |
| **41** | Lower-Dose Zinc for Childhood Diarrhea - A Randomized, Multicenter Trial | Children | Hb,hct, rdw, mcv, mchc, mpv Ferritin, sftr, znpp, zn | Lower doses of zinc had noninferior efficacy for the treatment of diarrhea in children and were associated with less vomiting than the standard 20-mg dose | Dhingra U et al (39) |
| **42** | SAZICMR2010 | Children | Hb, rdw, mcv, mchc, Ferritin, sftr, crp, agp |  | Not published |
| **43** | GRBKAR2018 | Pregnant women | Hb | Mothers who consumed Hot Cooked Meal at Anganwadi for >75 days had: • improved weight gain (total average from 1st to 3rd trimester 10.27 kgs.) • an increase in hemoglobin (average 0.52 percent from 1st to 3rd trimester) delivered babies with nearly 10 grams higher birth weight (2.84 v/s 2.74 kg) • Six percent decrease in symptoms of depression | Kachwaha S et al (40)  Giridhara RB (41) |

**References :**

1. Hirve S, Bhave S, Bavdekar A, Naik S, Pandit A, Schauer C, et al. Low dose ’Sprinkles’-- an innovative approach to treat iron deficiency anemia in infants and young children. Indian Pediatr. 2007 Feb;44(2):91–100.

2. Muthayya S, Thankachan P, Hirve S, Amalrajan V, Thomas T, Lubree H, et al. Iron fortification of whole wheat flour reduces iron deficiency and iron deficiency anemia and increases body iron stores in Indian school-aged children. J Nutr. 2012 Nov;142(11):1997–2003.

3. Neogi SB, Devasenapathy N, Singh R, Bhushan H, Shah D, Divakar H, et al. Safety and effectiveness of intravenous iron sucrose versus standard oral iron therapy in pregnant women with moderate-to-severe anaemia in India: a multicentre, open-label, phase 3, randomised, controlled trial. Lancet Glob Health. 2019 Dec;7(12):e1706–16.

4. Neogi SB, Negandhi H, Kar R, Bhattacharya M, Sen R, Varma N, et al. Diagnostic accuracy of haemoglobin colour strip (HCS-HLL), a digital haemoglobinometer (TrueHb) and a non-invasive device (TouchHb) for screening patients with anaemia. J Clin Pathol. 2016 Feb;69(2):164–70.

5. Neogi SB, Sharma J, Pandey S, Zaidi N, Bhattacharya M, Kar R, et al. Diagnostic accuracy of point-of-care devices for detection of anemia in community settings in India. BMC Health Services Research. 2020 May 26;20(1):468.

6. Kusneniwar G, Whelan RM, Betha K, Robertson JM, Ramidi PR, Balasubramanian K, et al. Cohort Profile: The Longitudinal Indian Family hEalth (LIFE) Pilot Study, Telangana State, India. Int J Epidemiol. 2017 Jun;46(3):788–789j.

7. Nagpal J, Sachdev HPS, Singh T, Mallika V. A randomized placebo-controlled trial of iron supplementation in breastfed young infants initiated on complementary feeding: effect on haematological status. J Health Popul Nutr. 2004 Jun;22(2):203–11.

8. Thiruvengadam R, Ayushi, Murugesan DR, Desiraju BK, Misra S, Sharma D, et al. Incidence of and risk factors for small vulnerable newborns in north India: a secondary analysis of a prospective pregnancy cohort. The Lancet Global Health. 2024 Aug 1;12(8):e1261–77.

9. Bhatnagar S, Majumder PP, Salunke DM, Interdisciplinary Group for Advanced Research on Birth Outcomes—DBT India Initiative (GARBH-Ini). A Pregnancy Cohort to Study Multidimensional Correlates of Preterm Birth in India: Study Design, Implementation, and Baseline Characteristics of the Participants. Am J Epidemiol. 2019 Apr 1;188(4):621–31.

10. Yajnik CS, Fall CHD, Coyaji KJ, Hirve SS, Rao S, Barker DJP, et al. Neonatal anthropometry: the thin-fat Indian baby. The Pune Maternal Nutrition Study. Int J Obes Relat Metab Disord. 2003 Feb;27(2):173–80.

11. Yajnik CS, Bandopadhyay S, Bhalerao A, Bhat DS, Phatak SB, Wagh RH, et al. Poor In Utero Growth, and Reduced β-Cell Compensation and High Fasting Glucose From Childhood, Are Harbingers of Glucose Intolerance in Young Indians. Diabetes Care. 2021 Dec;44(12):2747–57.

12. Rao S, Yajnik CS, Kanade A, Fall CH, Margetts BM, Jackson AA, et al. Intake of micronutrient-rich foods in rural Indian mothers is associated with the size of their babies at birth: Pune Maternal Nutrition Study. J Nutr. 2001 Apr;131(4):1217–24.

13. Babu GR, Murthy G, Deepa R, Yamuna null, Prafulla null, Kumar HK, et al. Maternal antecedents of adiposity and studying the transgenerational role of hyperglycemia and insulin (MAASTHI): a prospective cohort study : Protocol of birth cohort at Bangalore, India. BMC Pregnancy Childbirth. 2016 Oct 14;16(1):311.

14. Babu GR, Deepa R, Lewis MG, Lobo E, Krishnan A, Ana Y, et al. Do Gestational Obesity and Gestational Diabetes Have an Independent Effect on Neonatal Adiposity? Results of Mediation Analysis from a Cohort Study in South India. Clin Epidemiol. 2019;11:1067–80.

15. Babu GR, Murthy GVS, Reddy Y, Deepa R, Yamuna A, Prafulla S, et al. Small for gestational age babies and depressive symptoms of mothers during pregnancy: Results from a birth cohort in India. Wellcome Open Res. 2018;3:76.

16. Lobo E, Ana Y, Deepa R, Shriyan P, Sindhu ND, Karthik M, et al. Cohort profile: maternal antecedents of adiposity and studying the transgenerational role of hyperglycaemia and insulin (MAASTHI). BMJ Open. 2022 Sep 21;12(9):e063794.

17. Lobo E, R D, Mandal S, Menon JS, Roy A, Dixit S, et al. Protocol of the Nutritional, Psychosocial, and Environmental Determinants of Neurodevelopment and Child Mental Health (COINCIDE) study. Wellcome Open Res. 2024;9:486.

18. Dhurde VS, Patel AB, Locks LM, Hibberd PL. Anaemia prevalence, its determinants and profile of micronutrient status among rural school adolescent girls aged 14–19 years: a cross-sectional study in Nagpur district, Maharashtra, India. Public Health Nutr. 27(1):e248.

19. Goudar SS, Carlo WA, McClure EM, Pasha O, Patel A, Esamai F, et al. The Maternal and Newborn Health Registry Study of the Global Network for Women’s and Children’s Health Research. Int J Gynaecol Obstet. 2012 Sep;118(3):190–3.

20. Derman RJ, Goudar SS, Thind S, Bhandari S, Aghai Z, Auerbach M, et al. RAPIDIRON: Reducing Anaemia in Pregnancy in India-a 3-arm, randomized-controlled trial comparing the effectiveness of oral iron with single-dose intravenous iron in the treatment of iron deficiency anaemia in pregnant women and reducing low birth weight deliveries. Trials. 2021 Sep 23;22(1):649.

21. Taneja S, Chowdhury R, Dhabhai N, Upadhyay RP, Mazumder S, Sharma S, et al. Impact of a package of health, nutrition, psychosocial support, and WaSH interventions delivered during preconception, pregnancy, and early childhood periods on birth outcomes and on linear growth at 24 months of age: factorial, individually randomised controlled trial. BMJ. 2022 Oct 26;379:e072046.

22. Dwarkanath P, Muhihi A, Sudfeld CR, Wylie BJ, Wang M, Perumal N, et al. Two Randomized Trials of Low-Dose Calcium Supplementation in Pregnancy. New England Journal of Medicine. 2024 Jan 10;390(2):143–53.

23. Taneja S, Upadhyay RP, Chowdhury R, Kurpad AV, Bhardwaj H, Kumar T, et al. Impact of nutritional interventions among lactating mothers on the growth of their infants in the first 6 months of life: a randomized controlled trial in Delhi, India. The American Journal of Clinical Nutrition. 2021 Apr 1;113(4):884–94.

24. Maitri V, Tushar D, Dhiren M, Shrey D, Gayatri D. A Hospital Based Study to Access the Usefulness of Using the Combination Regime of the Ferric Carboxy Maltose, Vitamin B12 and Folic Acid as a Treatment of Severe Anemia among Pregnant Women in a Rural Tribal Community of South Gujarat. Indian J Community Med. 2022;47(3):437–40.

25. Taneja S, Upadhyay RP, Chowdhury R, Kurpad AV, Bhardwaj H, Kumar T, et al. Impact of supplementation with milk–cereal mix during 6–12 months of age on growth at 12 months: a 3-arm randomized controlled trial in Delhi, India. The American Journal of Clinical Nutrition. 2022 Jan 1;115(1):83–93.

26. Wadhwani NS, Sundrani DP, Wagh GN, Mehendale SS, Tipnis MM, Joshi PC, et al. The REVAMP study: research exploring various aspects and mechanisms in preeclampsia: study protocol. BMC Pregnancy Childbirth. 2019 Aug 23;19(1):308.

27. Ghosh S, Laxmaiah A, Chandak GR, Meshram II, Raman R, Sengupta S, et al. Anaemia and iron deficiency in India: a venous blood-based survey of adolescents, adults, and the elderly in eight states. Eur J Clin Nutr. 2025 Jan 8;

28. Gupta A, Sachdev HS, Kapil U, Prakash S, Pandey RM, Lal PR. Etiology of Mild and Moderate Anaemia Among Rural Adolescent Girls in India. Indian J Hematol Blood Transfus. 2024 Apr;40(2):255–60.

29. Gupta A, Sachdev HS, Kapil U, Prakash S, Pandey RM, Sati HC, et al. Characterisation of anaemia amongst school going adolescent girls in rural Haryana, India. Public Health Nutr. 2022 Jan 24;25(12):1–10.

30. Gupta A, Kant S, Ramakrishnan L, Pandey RM, Khandelwal R, Kapil U, et al. Impact of daily-supervised administration of a package of iron and folic acid and vitamin B12 on hemoglobin levels among adolescent girls (12-19 years): a cluster randomized control trial. Eur J Clin Nutr. 2021 Nov;75(11):1588–97.

31. Soni J, Saxena D, Saha S, Qureshi A, Patel B, Patel F, et al. Impact of Ayurvedic Interventions to improve the Nutritional Status among Adolescent Girls and Children under-five years in Devbhumi Dwarka district of Gujarat. Journal of Ayurveda and Integrated Medical Sciences. 2022 Dec 8;7(10):133–43.

32. Saha S, Puwar T, Shah K, Pandya A, Wanjari MB, Saxena D. Non-iron Deficiency Anemia in Rural Indian Women: A Cross-Sectional Study. Cureus. 14(8):e28565.

33. Palika R, Dasi T, Ghosh S, Peter R, Parasannanavar DJ, Pradhan AS, et al. Efficacy of iron-folic acid treatment for reducing anemia prevalence and improving iron status in women of reproductive age: A one-year longitudinal study. Clinical Nutrition ESPEN. 2022 Jun 1;49:390–7.

34. Gupta Bansal P, Singh Toteja G, Bhatia N, Kishore Vikram N, Siddhu A, Kumar Garg A, et al. Deficiencies of Serum Ferritin and Vitamin B12, but not Folate, are Common in Adolescent Girls Residing in a Slum in Delhi. Int J Vitam Nutr Res. 2015;85(1–2):14–22.

35. Sazawal S, Dhingra U, Hiremath G, Sarkar A, Dhingra P, Dutta A, Verma P, Menon VP, Black RE. Prebiotic and probiotic fortified milk in prevention of morbidities among children: community-based, randomized, double-blind, controlled trial. PLoS One. 2010 Aug 13;5(8):e12164. doi: 10.1371/journal.pone.0012164. PMID: 20730056; PMCID: PMC2921405.

36. Sazawal S, Dhingra U, Hiremath G, Sarkar A, Dhingra P, Dutta A, Menon VP, Black RE. Effects of Bifidobacterium lactis HN019 and prebiotic oligosaccharide added to milk on iron status, anemia, and growth among children 1 to 4 years old. J Pediatr Gastroenterol Nutr. 2010 Sep;51(3):341-6. doi: 10.1097/MPG.0b013e3181d98e45. PMID: 20601905.

37. Sazawal S, Dhingra U, Dhingra P, Dutta A, Deb S, Kumar J, Devi P, Prakash A. Efficacy of high zinc biofortified wheat in improvement of micronutrient status, and prevention of morbidity among preschool children and women - a double masked, randomized, controlled trial. Nutr J. 2018 Sep 15;17(1):86. doi: 10.1186/s12937-018-0391-5. PMID: 30219062; PMCID: PMC6139156.

38. Somji SS, Dhingra P, Dhingra U, Dutta A, Devi P, Kumar J, Deb S, Semwal OP, Sazawal S, Manji K, Kisenge R, Bakari M, Aboud S, Liu E, Sudfeld C, Duggan CP, Ashorn P, Bahl R, Simon JL. Effect of dose reduction of supplemental zinc for childhood diarrhoea: study protocol for a double-masked, randomised controlled trial in India and Tanzania. BMJ Paediatr Open. 2019 Apr 24;3(1):e000460. doi: 10.1136/bmjpo-2019-000460. Erratum in: BMJ Paediatr Open. 2020 Oct 12;4(1):e000460corr1. doi: 10.1136/bmjpo-2019-000460corr1. PMID: 31206083; PMCID: PMC6542451.

39. Dhingra U, Kisenge R, Sudfeld CR, Dhingra P, Somji S, Dutta A, Bakari M, Deb S, Devi P, Liu E, Chauhan A, Kumar J, Semwal OP, Aboud S, Bahl R, Ashorn P, Simon J, Duggan CP, Sazawal S, Manji K. Lower-Dose Zinc for Childhood Diarrhea - A Randomized, Multicenter Trial. N Engl J Med. 2020 Sep 24;383(13):1231-1241. doi: 10.1056/NEJMoa1915905. PMID: 32966722; PMCID: PMC7466932.

40. Kachwaha, S., Avula, R., Menon, P., Sethi, V., Joe, W., & Laxmaiah, A. (2021). Improving maternal nutrition in India through integrated hot-cooked meal programs: A review of implementation evidence.

41. https://www.indiascienceandtechnology.gov.in/research/evaluating-effect-one-full-meal-day-pregnant-and-lactating-women-feel
